# Notes on New Drugs and Preparations for the Sick

**Published:** 1902-06

**Authors:** 


### Notes on New Drugs and Preparations for the Sick.

**Tabloids.** — Burroughs, Wellcome, & Co., London. — 1. **Tabloid Hydrarg. Perchlor. et Potass. Iodid. Hydrarg. Perchlor.,** gr. $\frac{1}{16}$; **Potass. Iodid.,** grs. v.—Cases frequently occur in which the continued administration of mercury with iodide of potassium is essential. Mixtures, owing to their inconvenience and bulk, are unsuitable in many instances, and patients neglect to take doses regularly. The compactness of the "Tabloid" products permits a supply to be carried in the waistcoat pocket, and enables the treatment to be continued with regularity, and without being irksome to the patient. The drugs employed in the manufacture of the "Tabloid" products are of the highest quality, and the dose of gr. $\frac{1}{16}$ of mercury perchloride is equivalent to one drachm of the Liquor Hydrargyri Perchloridi.

2. **Tabloid Aspirin,** grs. v.—Aspirin is an acetyl salicylic acid, and is held to possess advantages over salicylic acid, and the salicylates, especially in the treatment of conditions associated with rheumatism. From grs. v. to grs. xv. are recommended for a dose, which should be repeated at short intervals.

We have been rather disappointed in this drug. Like the salicylates, it must be given in full doses; and when so given it produces very similar effects, both good and bad. It is difficult to differentiate between the action of the two, if given in similar doses.

3. **Tabloid Ophthalmic Atropine Sulphate,** gr. $\frac{1}{600}$.—"Tabloid" Ophthalmic Products possess obvious advantages over other methods of ophthalmic medication. They are designed to avoid the objections attendant upon the use of stock solutions which are apt to decompose. Being immediately soluble upon the conjunctiva and of high therapeutic efficiency, "Tabloid" Ophthalmic Atropine Sulphate will be found of great service.

4. **Soloid Nitro-propiol**, gr. ¼.—Nitro-propiol is of special value for confirming the presence of glucose in urine, when indicated by other reagents, such as Fehling's solution, inasmuch as the reaction is not influenced by uric acid, creatinine, glucuronic acid, bile colouring, or by bodies which may be contained in the urine after the administration of certain drugs.

To apply the test, mix 10 minims of the suspected urine with ʒiij. (10 c.c.) of water. Then add one "Soloid" Nitro-propiol and boil. If glucose be present the liquid will assume a blue colour, owing to the formation of indigo blue. This colour may appear quickly, but usually only after boiling for about three to five minutes.

The "Soloid" product renders the application of this test a simple matter, which will often be found useful.

**Enule Rectal Suppositories. Morphine Hydrochloride,** gr. ¼; **Extract of Belladonna,** gr. ½.—"Enule" Rectal Suppositories possess conspicuous advantages. Their improved shape makes them easy of introduction, and prevents their expulsion. Each is enclosed in a hermatically-sealed sheath of pure tinfoil, which prevents the possibility of contamination, and preserves from deterioration. The active principles are evenly diffused, and the dosage is of high accuracy.

"Enule" Morphine and Belladonna Suppositories are sedative and anodyne; they are of great use in relieving pain in irritable conditions of the rectum generally, in cancer of the lower bowel, in prostatitis, cystitis, and also in conditions of the deep urethra associated with pain.

**Soloid Microscopic Stains.**—B., W. & Co.—The following stains are now obtainable in this convenient form: Bismarck Brown, Borax Methylene Blue, Eosin, Eosin Methylene Blue, Louis Jenner, Fuchsine, Gentian Violet, Gram's Iodine Sol., Hæmatoxylin, Hæmatoxylin (Delafield), Methylene Blue, Methylene Violet, Thionine Blue. Solutions are readily prepared according to directions given. The sample submitted to us of Louis Jenner's stain has been given an extensive trial. In its favour is its very convenient form, especially for those who are not constantly using the stain. Against it we find, even after considerable trouble in powdering it, it is dissolved with considerable difficulty, so that to make a stain of the correct strength is not easy. The red corpuscles are nicely stained by the Eosin, but the white, although the nuclei show fairly well, do not leave their granules sufficiently well brought out to be of use in differential blood counts.

**Thyroglandin (Stanford).**—EVANS, LESCHER & WEBB, London.—This brown powder contains the active principles of the thyroid gland of the sheep. It is supplied in powder, pilules and pellets, and is four times the strength of the fresh gland. It is believed to contain the Iodoglobulin and Thyroiodin, which are said to be the active principles of the gland: it gives the xanthoproteic tests strongly, so that it is apparently an animal extract, but careful examination has failed to reveal the presence of Iodin, a substance difficult of detection when present in only small quantity (*Stanley Kent*).

**Ferri Alginas.—Alginoid Iron (Stanford).**—This brown insoluble powder is a combination of Iron and Alginic Acid, containing 10.97 % of Ferrum. It is soluble in Hydrochloric Acid, and gives the reaction for Iron in the Ferric state (*Stanley Kent*). The proprietors claim for it that it resists gastric digestion, but we question if this statement will bear criticism. The powder containing a considerable quantity of Iron in organic combination, should be very useful where the inorganic preparations are not desirable.

---

**Palmiacol, in Perles.**—THE TROMMER COMPANY.—This composition is given as $C_{23} H_{40} O_2$. We are told that the drug is more effectual than Guaiacol or Creasote on account of its non-irritating properties, but we are not informed as to its origin or mode of manufacture. Testimonials from many medical men in the United States state that it gives excellent results in catarrhal and tubercular conditions of the respiratory tract.

---

**Liquor Ipecac. Alb.**—(MATTHEWS, Clifton).—The range of usefulness of Ipecacuanha in practice as an expectorant is minimised by the depressing and emetic action of the cephæline and psychatrine, which are in association with the emetine. This purified preparation of Ipecacuanha contains only the emetine, and is free from the other alkaloids, and is therefore a distinct advance in pharmacy of much clinical value. We have found it is well tolerated in doses which, if given in the form of the Vinum Ipecac. would produce nausea; the dose and strength being the same in both preparations.

---

**Chocolate-coated Tablets.**—PARKE, DAVIS, & Co., London.—A very comprehensive list of these has been prepared, and it is claimed for them that they have additional permanence in consequence of the protection of the medicaments from the air, and that they have an improved appearance and greater palatability. The variety prepared appears to be such as should satisfy all the present demands and desires of mankind If these are accessible, there can be little need for anything more.

